# Fluid bolus therapy is a medical therapy or a diagnostic method?

**DOI:** 10.1186/s13054-015-1078-3

**Published:** 2015-10-13

**Authors:** Huaiwu He, Dawei Liu

**Affiliations:** Department of Critical Care Medicine, Peking Union Medical College Hospital, Chinese Academy of Medical Sciences, 1 Shuaifuyuan, Dongcheng District Beijing, China

We read with interest the recently published article about the physiological changes after fluid bolus therapy (FBT) in *Critical Care* by Glassford et al. [1]. We are concerned about the concept of FBT in this study.

The authors claim that alternative interventions to FBT may include a diagnostic low-volume FBT, classic fluid challenge, low-volume FBT and low dose vasopressor therapy, or cardiac output-guided therapy. So, FBT could be interpreted as a treatment and a diagnostic method for hypovolemia. We thought this interpretation would cause misunderstanding about FBT. FBT is commonly used to assess fluid responsiveness in hemodynamic management, which is also called ‘fluid challenge’ [2]. FBT essentially helps physicians to quickly make decisions regarding fluid management. So, when hypovolemia has been previously definitively diagnosed, it might be improper to define a bolus of fluid such as FBT. We believe that FBT is mainly a diagnostic method and not a method of therapy. We acknowledge that FBT could also be interpreted as a special mode of fluid infusion, but this point is unclear in the study by Glassford et al.

FBT resulted in a positive outcome in only about 50 % of cases in the ICU [3]. In other words, FBT should be avoided in half of critically ill patients. So, we think investigations should focus on how to reduce unnecessary FBT but not the physiological effects of FBT over 2 to 4 h.

## Abbreviation

FBT: Fluid bolus therapy
