# Supplementary material for: Fluid bolus therapy is a medical therapy or a diagnostic method?
Source: Crit Care. 2015 Oct 13;19:360. doi: 10.1186/s13054-015-1078-3 (PMC4603292; doi:10.1186/s13054-015-1078-3)
Supplement: Related texts

# Reply: Authors’ response

**Authors:** Neil J Glassford, Glenn M Eastwood, Rinaldo Bellomo

Liu and He ask if FBT is a medical therapy or diagnostic modality; the answer is, of course, context sensitive. In the context of the immediate management of the septic, critically ill patient, the administration of, often large, volumes of fluid in the form of FBT are an international therapeutic standard of care [4, 5]. FBT is also responsible for large proportions of therapeutic fluid administration in the critically ill [6] and other populations, including cardiac surgery patients [7]. In the context of this review, a fluid bolus was a defined volume of a defined fluid over a defined period of time [1].

We wonder if the confusion arises from one of the fundamental problems with research into fluid administration, fluid resuscitation and fluid responsiveness - the lack of fixed terminology. Moreover, fluid responsiveness is a complex topic, and not what we sought to investigate. We note that, in general, fluid responsiveness is identified retrospectively, and therefore tautologically. Indeed, from the evidence we were able to identify, there is very limited evidence for persistent physiological improvement even in patients identified as ‘fluid responsive’ [1].

We feel the question is not one of individual patient fluid responsiveness, but a larger one regarding the need to demonstrate the independent efficacy of FBT in improving patient outcomes. Such evidence is lacking in critically ill patients, whether fluid responsive or not.
